# Mechanical Modulation of S_0_–S_1_ and S_0_–T_1_ Energy Gaps of 11-*cis* and All-*trans* Retinal Schiff Bases

**DOI:** 10.1021/acs.jpcb.4c06631

**Published:** 2025-01-23

**Authors:** Alejandro Jodra, Luis Manuel Frutos

**Affiliations:** † Departamento de Química Analítica, Química Física e Ingeniería Química, 16720 Universidad de Alcalá, Alcalá de Henares, Madrid E-28871, Spain; ‡ Instituto de Investigación Química ‘‘Andrés M. del Río’’, Universidad de Alcalá, Alcalá de Henares, Madrid E-28871, Spain

## Abstract

The retinal Schiff base is a chromophore of significant biological relevance, as it is responsible for capturing sunlight in rhodopsins, which are photoactive proteins found in various living organisms. Additionally, this chromophore is subjected to various mechanical forces in different proteins, which alter its structure and, consequently, its properties. To thoroughly understand the mechanical response limits of the retinal excitation energy, a simple first-order formalism has been developed to quantify the chromophore’s optimal mechanical response to applied external forces (on the order of tens of pN). Additionally, the response to larger forces is analyzed by using an algorithm to explore the potential energy surfaces. It can be concluded that the retinal Schiff base exhibits a significant mechanical response and that the optimal forces and displacements involve certain coordinates typically of low frequency, showing differences between the S_1_ and T_1_ states, as well as between the 11-*cis* and all-*trans* isomers. Additionally, the possibility of mechanically modulating the bond length alternation using mechanical forces is ruled out.

## Graphical abstract

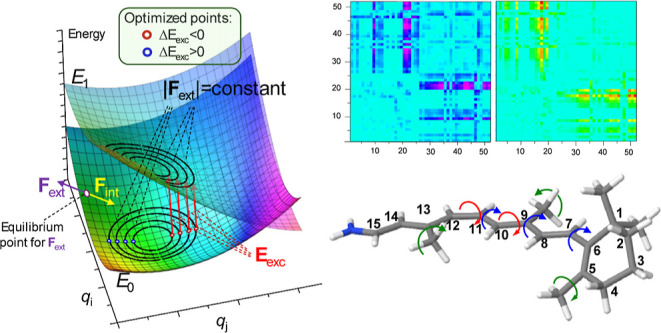

## Introduction

The use of mechanical forces to control photochemical and photophysical processes in molecular systems has been explored in recent years.^1^ It has been demonstrated that applying mechanical forces via molecular force probes can modulate photochemical properties such as fluorescence yield, excited-state lifetime, and photoisomerization quantum yield in stilbenes.^2^ Similarly, exerting tensile forces on a retinal chromophore model increases the *trans*-to-*cis* photoisomerization quantum yield.^3^ Computational methods and models have been developed to study the control of photophysical properties like absorption spectra,^4^ as well as photochemical reactivity.^5,6^ These mechanochemical approaches offer a path to control molecular photoreactivity and may guide through the design of novel mechano-responsive chromophores.^7^

The retinal Schiff base is a chromophore that is subjected to mechanical forces in different contexts, especially within rhodopsins,^8^ which are photoactive proteins with numerous functions in different organisms. Understanding how this chromophore responds to mechanical forces is particularly interesting due to its biological, technological, and scientific relevance.

Researchers have extensively studied the structure, properties, and behavior of retinal in different environments including inside rhodopsin proteins, through both experimental and theoretical methods.^9^ One particular approach that has been highly beneficial is the use of multiconfigurational methods within a QM/MM framework.^10^ Notably, the research led by Andruniów et al. has significantly contributed to unveiling details about the structure,^11^ optical properties,^12^ and dynamics of these systems.^13^ The control of excitation energy within the protein environment, specifically the opsin cavity, involves two main components: structural and electrostatic.^14^ The structural aspect involves steric interactions from the amino acids within the protein pocket, while the electrostatic aspect involves interactions with ions and amino acids that have partial charges.

The protonated retinal Schiff base (RET in the following), the chromophore inside rhodopsin, exhibits complex excited-state dynamics in solution. Ultrafast spectroscopy reveals multiple decay components in the excited state, with lifetimes ranging from femtoseconds to picoseconds.^15^ The absorption spectrum RET in vacuo shows a maximum at 610 nm, providing a reference for understanding spectral tuning in rhodopsins.^16^ This gas-phase measurement demonstrates that protein environments in rhodopsins actually blue-shift the absorption, contrary to previous comparisons with solvents. The UV–visible absorption spectrum of all-*trans* and 11-*cis* RET in the gas phase has been studied.^17^ External positive charges significantly influence the absorption maximum of RET, especially when the interaction between the external charge and its counterion is weakened.^18^ In this regard, multiconfigurational perturbation theory calculations have achieved quantitative agreement with experimental absorption maxima for protonated and deprotonated Schiff bases of all-*trans*- and 11-*cis*-retinal, covering a wavelength range from 610 to 353 nm.^19^

The role of singlet and triplet excitations in the retinal chromophore of rhodopsin has been extensively studied through computational and experimental approaches. The population of triplet states is proposed to arise from small S_1_–T_1_ energy gaps and the activation of specific vibrational modes.^20^ Energy-transfer techniques have been used to populate the triplet state in bacteriorhodopsin and model compounds.^21^ Moreover, the crossing between the S_0_ and T_1_ states of retinal in rhodopsin has been identified as a potentially efficient pathway for isomerization following T_1_ excitation.^22^ Consequently, T_1_ excitation plays a critical role in both direct and sensitized triplet population mechanisms in the retinal chromophore, significantly influencing the dynamics of rhodopsin’s visual cycle.

In this work, we present an investigation on the mechanical response of RET chromophore (i.e., 11-*cis* and all-*trans* retinal protonated Schiff base) to explore the limits of the mechanical tuning in the energy gaps corresponding to S_0_ → S_1_ and S_0_ → T_1_ electronic states. By using different mechanochemical approaches, we identify the optimal response of these energies to the action of mechanical forces, providing the boundaries of mechanical modulation of absorption spectra. Additionally, the relevant coordinates involved in this mechanical modulation are analyzed along with the possible implementations of the mechanical forces like using force pairs.

## Methodology

Electronic structure calculations were performed using the CAM-B3LYP functional with the 6-311+G* basis set, as implemented in Gaussian 16.^23^ This method was used for determining mechanical responses using analytical surfaces as well as for exploring the complete potential energy surfaces (PES). For the excited-state calculations, time-dependent density functional theory was employed to investigate both the first singlet (S_1_) and the first triplet (T_1_) excited states. These calculations employ analytical gradients and Hessians, which are crucial for applying the different mechanochemical models in understanding the photophysical behavior of the system under investigation.

To calibrate our computational approach, the linear response approximation was benchmarked using the complete active space self-consistent field with a (12,12) active space. This method provided mechanical response vectors and energetic profiles that were closely aligned with those obtained via DFT, thus validating the accuracy and reliability of our chosen computational approach. Nevertheless, the CAM-B3LYP method has been previously proved to produce very accurate results in these chromophores even for configurational regions relatively far from the Franck–Condon (FC) region.^24^

The exploration of the PES—ground and excited states—was performed using the larger force minimum gradient (LFMG) algorithm.^25^ Additionally, calculations involving analytical PES were in part performed with MATLAB^26^ and in part with our own developed codes.

## Results

In the following, we analyze the mechanical response of the S_0_–S_1_ and S_0_–T_1_ energy gaps in 11-*cis* and all-*trans* retinal. First, we apply a simple first-order approach using analytical PES to explore the mechanical limits of energy gap tuning, obtaining the optimal forces and the structural changes derived from them, controlling the vertical excitation energy. Additionally, the applications of force pairs are analyzed as a practical means to implement mechanical forces, and the corresponding variation of the energy gap is predicted accordingly. This approximation is only valid for small force magnitudes; therefore, we finally make use of the LFMG algorithm to explore the exact mechanical response and the identification of the optimal forces as a function of energy gap variation.

### Effect of an External Force on the Excitation Energy

In order to describe the mechanical behavior of the retinal chromophore in a first approach (i.e., valid for low force magnitudes), a second-order approach in the PES can be assumed.

The energy of the ground state, “0”, is therefore

1 $$E_{0}\left(q\right)=E_{0}\left(0\right)+\frac{1}{2}q^{T}H_{0}q$$

where ***q*** = 0 corresponds to the ground-state equilibrium structure of the chromophore. The application of a given mechanical force, ***F***_ext_, affects the equilibrium structure of the system, which is now given by the point where internal and external forces cancel out (***F***_ext_ + ***F***_int_ = 0, see Figure 1). Since the internal forces are given by

2 $$F_{\mathrm{int}}=-H_{0}q$$

**Figure 1:**
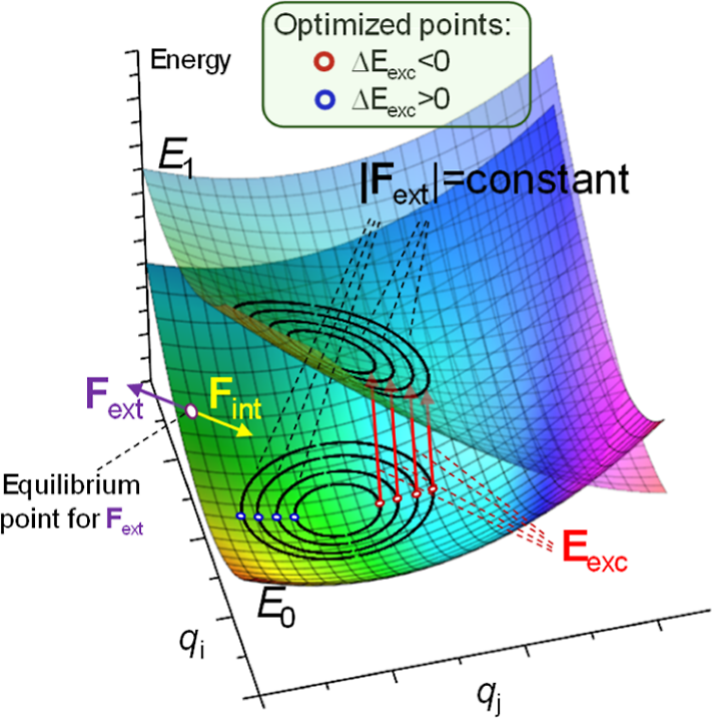
Schematic representation of two PES (*E*_0_ and *E*_1_) where the color map corresponds to the vertical energy difference. An equilibrium point under the action of an external force ***F***_ext_ is reached when external and internal forces are opposed: ***F***_ext_ = −***F***_int_. Isocontours of constant force magnitude in the *E*_0_ state are shown; inside this, hypercurves’ optimal points correspond to the largest increase or decrease in the energy gap.

The new equilibrium structure $q_{\text{eq}}^{F_{\text{ext}}}$ under the effect of the external forces, ***F***_ext_, is

3 $$q_{\text{eq}}^{F_{\text{ext}}}=H_{0}^{-1}F_{\text{ext}}$$

Second-order approximation of PES is the minimum necessary for the ground state because it provides the variation of the internal forces as a function of coordinates, and therefore, it is possible to predict the variation of the equilibrium geometry $q_{\text{eq}}^{F_{\text{ext}}}$ with the applied force.

Making the same approximation for an excited-state PES, “1”

4 $$E_{1}\left(q\right)=E_{1}\left(0\right)+q^{T}g_{1}+\frac{1}{2}q^{T}H_{1}q$$

where **g**_1_ and **H**_1_ are the gradient vector and Hessian matrix, respectively, evaluated for the ground-state equilibrium ***q*** = 0 configuration. The vertical excitation energy can therefore be expressed as a function of the nuclei configuration

5 $$E_{\text{exc}}\left(q\right)=E_{\text{exc}}\left(0\right)+q^{T}g_{1}+\frac{1}{2}q^{T}\left(H_{1}-H_{0}\right)q$$

where *E*_exc_(0) = *E*_1_(0) – *E*_0_(0) is the vertical excitation energy from the ground-state ***q*** = 0 structure. Therefore, since the application of an external force changes the equilibrium structure, the vertical excitation energy will also change

6 $$E_{\text{exc}}\left(q_{\text{eq}}^{F_{\text{ext}}}\right)=E_{\text{exc}}\left(0\right)+{\left(q_{\text{eq}}^{F_{\text{ext}}}\right)}^{T}g_{1}+\frac{1}{2}{\left(q_{\text{eq}}^{F_{\text{ext}}}\right)}^{T}\left(H_{1}-H_{0}\right)q_{\text{eq}}^{F_{\text{ext}}}$$

### Optimal Force for Tuning the Excitation Energy: First-Order Approach

Among all of the forces that can be applied to a molecular system, those that are optimal are of particular interest because they produce the largest variation in excitation energy with the smallest possible force magnitude. These forces are crucial, as they define the limits of the mechanical response of the excitation energy in a chromophore. To achieve a first-order approach, it is necessary to expand the PES of the ground state up to second order, which permits us to describe the applied force as a function of the structure (see above). Meanwhile, the energy difference can be approximated to the first order, which constitutes the simplest possible description of excitation energy with coordinates.

At this point, it is necessary to minimize the applied external force with the restriction of reaching a given excitation energy variation. In order to do this, the Lagrange multiplier method is a straightforward mathematical tool

7 $$L_{F_{\text{ext}}}={∥F_{\text{ext}}∥}^{2}+\lambda \left(E_{\text{exc}}\left(q\right)-E_{\text{exc}}\left(0\right)-C\right)$$

where we have chosen ${∥F_{\text{ext}}∥}^{2}$ as the function to minimize instead of ∥ ***F***_ext_∥ for mathematical simplicity (avoiding the square root) and λ being the Lagrange multiplier and *C* a constant defining the shift in the excitation energy. Substituting for the mentioned approximations, eq 7 becomes

8 $$L_{F_{\text{ext}}}=q^{T}H_{0}^{2}q+\lambda \left(q^{T}g_{1}-C\right)$$

Optimizing the Lagrange function yields to

9 $$\nabla L_{F_{\text{ext}}}=2H_{0}^{2}q_{\text{opt}}^{F_{\text{ext}}}+\lambda g_{1}=0$$

Or equivalently

10 $$q_{\text{opt}}^{F_{\text{ext}}}=\frac{-\lambda }{2}H_{0}^{-2}g_{1}$$

It has to be noted that the Lagrange multiplier just defines the length of the geometry displacement vector, but not its direction; therefore, the $q_{\text{opt}}^{F_{\text{ext}}}$ optimal vector direction is proportional to

11 $$q_{\text{opt}}^{F_{\text{ext}}}\propto \pm H_{0}^{-2}g_{1}$$

where the two signs take into account the two possible variations in the excitation energy (i.e., increase or decrease). Considering the optimal displacement, the optimal force is given by

12 $$F_{\text{ext},\text{opt}}=\frac{-\lambda }{2}H_{0}^{-1}g_{1}$$

And the variation in the excitation energy ( $\Delta E_{\text{exc}}\left(q_{\text{opt}}^{F_{\text{ext}}}\right)\equiv E_{\text{exc}}\left(q_{\text{opt}}^{F_{\text{ext}}}\right)-E_{\text{exc}}\left(0\right)$ ) is equal to:

13 $$\Delta E_{\text{exc}}\left(q_{\text{opt}}^{F_{\text{ext}}}\right)=\frac{-\lambda }{2}g_{1}^{T}H_{0}^{-2}g_{1}$$

The first-order approach giving the optimal response of the chromophore excitation energy to applied force is therefore given by

14 $$\gamma_{F}\equiv \frac{\Delta E_{\text{exc}}\left(q_{\text{opt}}^{F_{\text{ext}}}\right)}{∥F_{\text{ext},\text{opt}}∥}=-{\left(g_{S_{1}}^{T}H_{S_{0}}^{-2}g_{S_{1}}\right)}^{1/2}$$

γ_F_ provides a measure (first approach) of the mechanical sensitivity of the chromophore to an external optimal force. It is measured in Energy/Force and delimitates the maximal response (i.e., increase and decrease) of the excitation energy as a function of the applied force magnitude.

### Optimal Force Using Complete PES

In order to go beyond the quadratic approach, it is possible to explore a complete PES without restrictions, locating optimal points for a wide range of forces. In order to do this, we have employed the LGMF algorithm (largest energy gap variation with minimal mechanical force) developed by us.^25^

This algorithm basically looks for the higher energy gap with the restriction of a constant force magnitude, i.e., ***F***_ext_ = *c*, with “*c*” a given constant. This constant is varied smoothly exploring the exact relation between the applied mechanical force and the optimal response in the energy gap.

### Mechanical Response of S_1_ and T_1_ Energy Gaps in Retinal

#### First-Order Approach

Applying the above approach to retinal chromophore, it is possible to identify the optimal forces (eq 12), the nuclear displacement according to that force (eq 11), and the response in terms of variation of the excitation energy per force unit (eq 14). This approach, even if valid only for relatively low forces, i.e., typically in the range of hundreds of pN where the linear approach remains valid, provides a first frame to evaluate the mechanical sensitivity of the chromophore to the vertical energy gap.

The mechanical sensitivity of the chromophore is higher for *cis*-RET­(S_1_) (γ_F_ = 0.0698 kcal·mol^–1^·pN^–1^), being lower for the *trans*-RET­(S_1_) state (γ_F_ = 0.0198 kcal·mol^–1^·pN^–1^), and similar intermediate values for the triplet state: *cis*-RET­(T_1_) (γ_F_ = 0.0370 kcal·mol^–1^·pN^–1^) and *trans*-RET­(T_1_) (γ_F_ = 0.0357 kcal·mol^–1^·pN^–1^). Quantitatively similar results are obtained when the vectors are determined at CASSCF­(12,12)/6-31G*: S_1_ and T_1_ gradient vectors are qualitatively equivalent to those obtained at CAM-B3LYP/6-311 + G**, and the first-order approach predictions are equivalent. The optimal mechanical variation of the wavelength with applied force is given in Figure 2.

**Figure 2:**
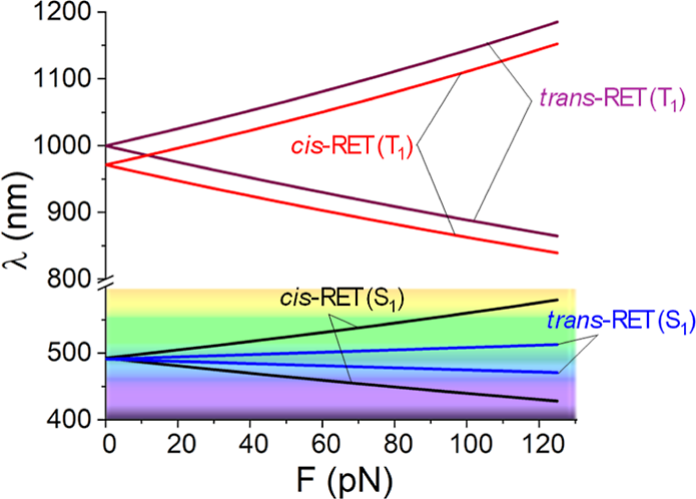
Variation in the vertical energy gap (wavelength in nm) as a function of the magnitude of the applied force (in pN) for *cis* and *trans* retinal and S_1_ and T_1_ states.

#### Optimal Force and Displacement Vectors

The following sections discuss the various contributions of the optimal force vectors obtained according to eq 12, as well as the structural displacements caused by the application of these forces (eq 11). In general, it should be noted that both vectors are qualitatively similar since the forces applied to specific nuclei induce displacements that are also qualitatively similar. However, it should be noted that due to the coupling between different coordinates, this relationship is qualitative, with both the components and their weights varying.

Furthermore, it is essential to highlight that, as a linear approximation, the same vectors (force and displacement) are responsible for both increasing and decreasing the energy gap between the states considered (what determines one or the other is the direction of the vector).

Figure 3 shows the displacements generated by the optimal force in the modulation of the S_0_–S_1_ and S_0_–T_1_ energy gaps in both *cis*-RET and *trans*-RET.

**Figure 3:**
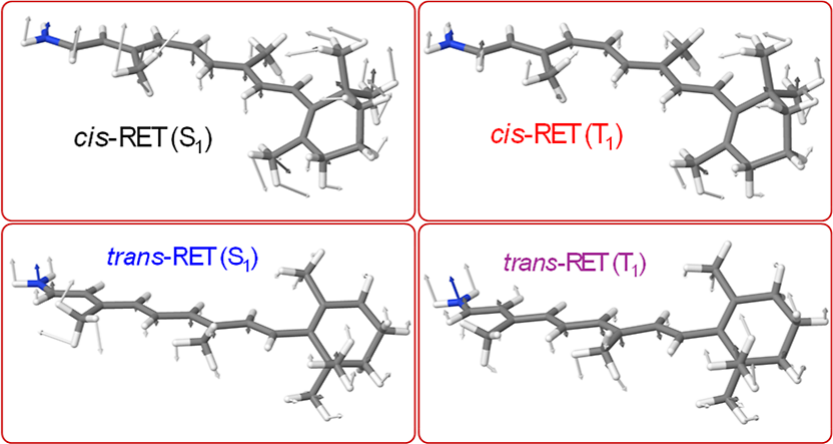
Coordinate displacement vectors that are the response to the application of the optimal forces obtained from eq 12.

In the case of the *cis* S_1_ and T_1_ states, it is important to note that the twisting of the methyl groups (see Figure 4), especially the methyl attached to C13, as well as the one attached to C5, has a significant contribution, as they are coupled to the torsions around the C11–C12 (11-*cis*) and C6–C7 (β-ionone) bonds, respectively. In fact, the dominant torsion in the case of the S_1_ state corresponds to the C11–C12 (11-*cis*) bond, which has a contribution about four times larger than the torsion around C6–C7 (β-ionone). Similarly, in the mechanical modulation of T_1_ energy, torsions around the C11–C12 and C6–C7 bonds also play a relevant role but this time with nearly equal weight. Additionally, the torsion around the single C8–C9 bond has slightly more weight than the previous torsions (6:4 ratio). Methyl torsions are also significant in this case.

**Figure 4:**
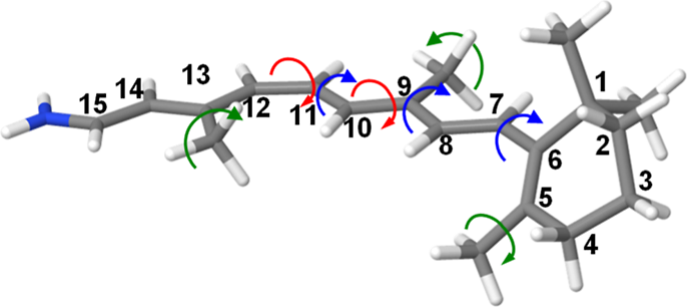
Structure of *cis*-retinal showing the numbering of carbon atoms. The methyl group torsions are indicated with green arrows, C=C torsions with red arrows, and C–C torsions with the blue ones.

In the case of *trans*-RET, the contribution of the torsion around the C11–C12 bond almost disappears, showing that the *cis* configuration of the double bond makes it mechanically sensitive in the modulation of S_1_ and T_1_ energies, whereas in the all-*trans* configuration, this preference does not exist. For S_1_, the largest contribution again comes from the methyl groups (especially the methyl group attached to C13). This movement is related to torsions around the single and double carbon–carbon bonds: C8–C9 and C9–C10 (in approximately 2:1 proportions). In this sense, the primary torsions are more likely to *cis*-RET (T_1_) than to *cis*-RET (S_1_). Last, for *trans*-RET (T_1_), the largest contributions are once more the rotation of the methyl attached to C13, as well as the torsions around single bonds C8–C9, C10–C11, and C12–C13 (in approximately 3:2:2 proportions). In this case, the low-frequency torsional movements associated with these dihedrals result in a significant contribution in varying the energy gap.

#### Force Pairs

An interesting approach for studying the mechanical response of a chromophore involves considering force pairs, which closely aligns with most experimental techniques. The procedure to computationally determine the mechanical response of the energy gap for a specific state is as follows: first, all possible different 1275 atom pairs are identified, and an external pulling force is applied to each pair sequentially. The new equilibrium geometry in response to the force pair is then determined for each pair. Finally, using the analytical PES (eq 6), the excitation energy is calculated.

To exemplify this procedure, we focus on *cis*-RET (S_1_), which is likely the most relevant isomer due to its significance in mammalian vision among other functions. The force pair matrices reveal the effective force pairs that modulate the S_0_–S_1_ energy gap (see Figure 5).

**Figure 5:**
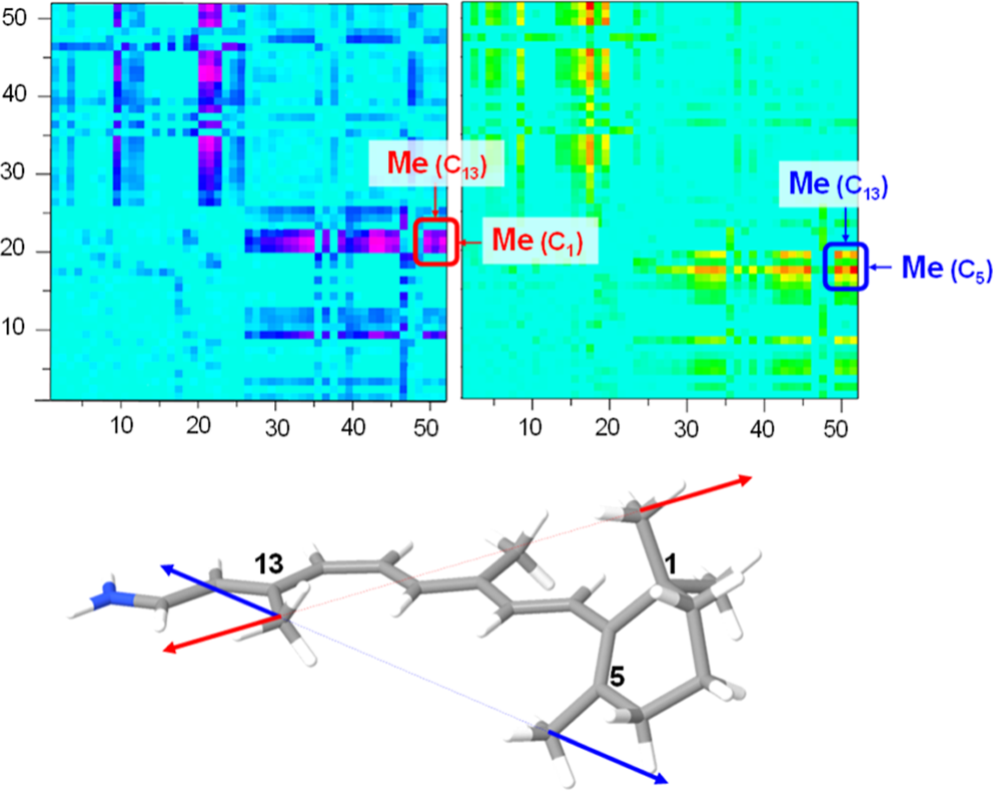
Force pair excitation energy matrices for S_0_–S_1_ energy gap in *cis*-RET. In the top-left panel, the negative variation of the excitation energy is shown (largest component due to the methyl in C13 and in C1), and the positive variation is shown in the upper-right panel (largest component due to the methyl in C1). In the bottom, the two optimal force pairs (excitation energy decrease, red, and increase, blue) for *cis*-RET are shown.

It is found that the most relevant force pairs involve methyl groups: one at C13 and the others linked to C5 or C1. These pulling forces induce torsion around the C11–C12 bond and also impact the torsion of the β-ionone ring. Specifically, a pulling force applied to the methyl carbon atoms linked to C5 and C13 causes the largest increase in the excitation energy, while the force pair between the carbon atom of the methyl group linked to C1 and the terminal N atom results in the largest decrease in the excitation energy. This observation aligns with the previously noted role of β-ionone ring torsion.^27^ In both scenarios, the torsion of the β-ionone ring is affected, decreasing in the first case and increasing in the second case, thereby causing the excitation energy to increase and decrease, respectively.

It is well known that bond length alternation (BLA) is a key structural parameter controlling the S0 → S1 excitation energy in 11-*cis* RET. The electrostatic environment of this chromophore inside the rhodopsin protein is known to efficiently control the excitation energy through variations in this coordinate.^12^ In this context, we analyzed the potential control of the BLA using external forces. To explore this possibility, we constructed the coordinate displacement vector associated with BLA, **q**_BLA_, and determined the external force that modifies BLA according to ***F***_ext_^BLA^ = ***H***_0_ ***q***_BLA_. We found that a significant mechanical response is unlikely, as large forces are required to affect this coordinate and, consequently, the excitation energy. Specifically, the mechanical response is limited to −0.25 kcal·mol^–1^ per nN, which is inefficient from a practical standpoint.

#### Complete PES

In order to go beyond the first-order approaches using analytical PES, it is possible to explore the complete PES in the different electronic states to find the optimal forces and displacements provoking the largest variations in the energy gap with the minimal force magnitudes. In order to do this, we used the largest energy gap variation with the minimal mechanical force (LGMF) algorithm as implemented by the authors.^25^

The mechanical response of *cis* and *trans* retinal in different states shows a qualitatively similar trend as that predicted with a linear approach. Nevertheless, there is overall different quantitative behavior. First, the average γ_F_ parameter takes similar values for all the cases (in kcal·mol^–1^·nN^–1^ units): $\gamma_{F,cis-\text{RET}\left(S_{1}\right)}=3.5$ , $\gamma_{F,trans-\text{RET}\left(S_{1}\right)}=3.1$ , $\gamma_{F,cis-\text{RET}\left(T_{1}\right)}=4.4$ , and $\gamma_{F,trans-\text{RET}\left(T_{1}\right)}=3.0$ (see Figure 6). These average values are taken from the exploration done up to about 0.5 nN force magnitude in all cases. The exploration of complete PES shows that the linear approach has some limitations for quantitative prediction of the mechanical response as it tends to overestimate the role of low-frequency modes like methyl torsions that initially (tens of pN range) have some impact in the excitation energy due to the coupling with torsions but that for larger forces (hundreds of pN) they tend to play a minor role.

**Figure 6:**
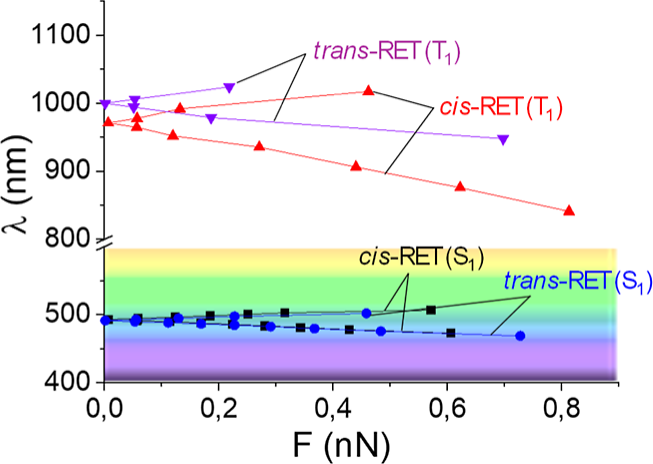
Mechanical response of energy gap wavelength λ­(nm) as a function of the optimal force magnitude (nN) for *cis* and *trans* retinal and S_1_ and T_1_ states. Complete PES have been used by applying the LGMF algorithm.

In order to clarify the distortions induced by the optimal forces for relatively large magnitudes, we have analyzed the optimal distortions for the larger forces applied (i.e., ca. 0.5 nN). First, it has to be noted that in all cases, the β-ionone torsion represents the largest contribution to this distortion (see Table 1). This coordinate has an equilibrium value of 42.2° for *cis* isomer and 41.5° for the all-*trans* isomer. In all the cases for 11-*cis* and all-*trans*, the application of the optimal force that increases the energy gap also increases this dihedral angle and vice versa.

**Table 1:** Relative Variation of the Coordinates by Applying Optimal Forces^a^

RET	Δ*E*_exc_	β-ionone	C8–C9	Me–C9
*cis*-**S**_**1**_	–	39.1 (−)	35.8 (+)	25.1 (−)
*cis*-**S**_**1**_	+	60.4 (+)	39.6 (−)	
*cis*-**T**_**1**_	–	56.8 (−)	29.7 (+)	
*cis*-**T**_**1**_	+	57.7 (+)	42.3 (−)	
*trans*-**S**_**1**_	–	37.5 (−)	31.3 (+)	31.3 (−)
*trans*-**S**_**1**_	+	56.1 (+)	43.9 (−)	
*trans*-**T**_**1**_	–	61.4 (−)	38.6 (+)	
*trans*-**T**_**1**_	+	57.8 (+)	42.2 (−)	

a Δ*E*_exc_ indicates the sign of the variation in the energy gap (i.e., “+” and “–” indicate the increase and decrease in the energy gap). The relative con weight (percentage) of the most relevant coordinate contributions is shown with an addition “+” or “–” sign indicating the direction of the torsion variation. Contributions smaller than 5% are not shown.

Another relevant mechanical coordinate is the C7–C8–C9–C10 torsion (C8–C9 in table A; see Figure 4 for numbering). This torsion is −177.2° for 11-*cis* RET and −177.4° for all-*trans* RET. A mechanical increase in the excitation energy (Δ*E*_exc_ > 0) implies a decrease in the torsion value (note that since it is initially a negative torsion, decreasing the angle implies larger absolute values of the torsion). Finally, a third relevant mechanical coordinate is the rotation of the methyl linked to carbon atom 9, which only plays an important role in *cis* (S_1_) and all-*trans*-(S_1_) when decreasing the energy gap. Rotation of the methyl in the direction indicated by the arrows in Figure 4 is responsible for the energy gap decrease in these two cases.

A direct comparison with the structure of retinal in rhodopsin proteins is not feasible since electrostatic and steric effects are inherently coupled, making it impossible to separate them in a straightforward manner. However, the obtained results can be qualitatively analyzed by considering previous studies that decompose these effects on the retinal chromophore under various environments, such as the gas phase, solvents, and inside rhodopsin. Previous studies agree with the critical role of torsional coordinates in controlling the excitation energies. Specifically, the torsion around the C11=C12 bond is consistently highlighted as the dominant coordinate in the 11-*cis* retinal, particularly in the modulation of the S_1_ state energy.^28^ Additionally, the β-ionone torsion has been identified as a key structural parameter to control the excitation energy in retinal,^29^ being proposed as a fine-tuning coordinate for blue shift in some rhodopsins.^30^ Our findings align with these observations, confirming the β-ionone torsion as a critical mechanical coordinate for modulating the excitation energy in both singlet and triplet states. They also highlight the importance of including low-frequency torsional modes, particularly for 11-*cis* retinal, in explaining the observed shifts in excitation energy.

On the other hand, the electrostatic interaction between the retinal Schiff base and Glu113 has been identified as a critical factor in fine-tuning the excitation spectrum through its effect on BLA coordinate.^31^ This agrees with our findings, which indicate that direct mechanical control of BLA is inefficient due to the unfeasibly large forces required to significantly modulate it.

## Conclusions

Using mechanochemical computational models, we demonstrate that 11-*cis* and all-*trans* retinal Schiff bases exhibit significant mechanical responses in their S_0_–S_1_ and S_0_–T_1_ energy gaps. The key coordinates involve three main torsions: one related to the β-ionone ring, another to the C7–C8–C9–C10 torsion, and a third to the Me–C9 torsion. These coordinates effectively control the energy gaps, allowing for an increase or decrease with a ratio of approximately 3–4 kcal·mol^–1^·nN^–1^. Additionally, tensile force pairs can be applied to 11-*cis* RET, resulting in a less efficient yet significant mechanical response, achieving about 1.4 kcal·mol^–1^·nN^–1^ for both decreasing and increasing the energy gap. While BLA has been identified as an important coordinate in controlling the energy gap in 11-*cis* RET, our analysis of mechanical induction of this distortion, particularly through the use of force pairs, reveals that it cannot be efficiently activated mechanically. Only minimal contributions with very weak mechanical responses are observed, effectively ruling out the mechanical activation of the BLA coordinate in 11-*cis* RET.

These findings have several potential applications in the design of retinal-based mechanosensors since the relevant mechanical response of retinal Schiff bases to specific torsions could be exploited to design molecular sensors that detect and quantify mechanical forces in biological systems. Additionally, the ability to control energy gaps through mechanical forces could lead to the development of molecular switches that respond to both light and mechanical stimuli, which are potentially useful in nanoscale devices or smart materials.
